# Electromagnetic Navigation Bronchoscopy Integrated Non-intubated Uniportal VATS in Localization and Resection of Pulmonary Nodules

**DOI:** 10.3389/fsurg.2022.872496

**Published:** 2022-04-05

**Authors:** Rui Wang, Yu Jiang, Jiaxi He, Yuechun Lin, Zhufeng Wang, Shuben Li

**Affiliations:** ^1^Department of Thoracic Surgery, The First Affiliated Hospital of Guangzhou Medical University, Guangzhou, China; ^2^China State Key Laboratory of Respiratory Disease, Guangzhou Institute of Respiratory Disease, Guangzhou, China

**Keywords:** pulmonary nodule, electromagnetic navigation bronchoscopy, non-intubated anesthesia, video-assisted thoracoscopic surgery, integrated operating room

## Abstract

**Background:**

With the development of computed tomography, the detection rate of pulmonary nodules is increasing. Accurate localization, minimally invasive resection, and rapid recovery are the most concentrated issues in modern thoracic surgery. However, some traditional procedures, including CT-guided localization and general intubated anesthesia, might prolong the operation and postoperative recovery. The integrated operating room provides a practical approach to achieve precise pulmonary nodule localization with real-time images using electromagnetic navigation bronchoscopy (ENB). Meanwhile, the minimally invasive video-assisted thoracoscopic surgery (VATS) under non-intubated anesthesia is also applied in the same place, enhancing operative efficiency and recovery after surgery.

**Method:**

The patients with pulmonary nodules resection who underwent nodules localization and uniportal VATS under non-intubated anesthesia in the integrated operating room between September 2018 and December 2021 were identified and collected. They all received ENB localization before uniportal VATS under non-intubated anesthesia, provided by the same group of anesthesiologists and surgeons. Perioperative data of patients were analyzed and evaluated to demonstrate the feasibility and efficiency of the procedure.

**Result:**

A total of 243 patients with 251 pulmonary nodules underwent ICG staining localization by ENB. The mean calibration time and navigation time were 0.91 ± 0.43 min and 10.56 ± 7.24 min, respectively. Overall, successful navigation occurred in 248 (98.80%) nodules. All patients received thoracoscopic surgery after localization, including wedge resection (231, 92.03%), segmentectomy (13, 5.18%), and lobectomy (7, 2.79%). All nodules were completely resected without serious complications. The mean postoperative hospital was 1.80 ± 0.83 days.

**Conclusion:**

ENB localization and nodules resection under non-intubated uniportal VATS in the integrated operating room provides a feasible and efficient approach to the pulmonary nodules patients, favoring the treatment precision and enhanced recovery.

## Introduction

With the wide use of chest computed tomography (CT), more potentially operable lung cancer patients can be identified at an early stage and receive timely surgical treatments. A study (1) pointed out that China has the highest incidence of lung cancer among Asian races. Therefore, a novel surgical approach for early and precise excision of pulmonary nodules is urgently needed. The lung resection can be performed in a less invasive way via video-assisted thoracoscopic surgery (VATS) with three-port incisions or even a single incision. Because of its advantages of high flexibility and minor trauma, VATS has become the preferred surgical method for pulmonary nodules resection (2, 3), including wedge resection, segmentectomy, and lobectomy.

More advanced strategies have improved surgical outcomes, including non-intubated anesthesia and electromagnetic navigation bronchoscopy (ENB). Non-intubated anesthesia is a new advanced strategy conducted by locoregional anesthesia with spontaneous ventilation without side effects of general anesthesia and one-lung ventilation. Compared with endotracheal intubation, which significantly reduced the incidence of postoperative complications, shortened the length of hospital stay (4), attenuated stress, inflammatory responses, and stimulation of cellular immune function (5), thus enhancing the rapid recovery. The lesion is usually located by CT or bronchoscopy in the clinic. Generally, the pulmonary nodules can be precisely localized by the traditional CT-guided method. However, complications such as hemorrhage, hematoma, and pneumothorax are likely to occur during the procedure (6, 7). The electromagnetic navigation bronchoscope may be a better alternative to solve these problems.

Given the merits of both uniportal VATS under non-intubated anesthesia and ENB, the concept of the integrated operating room was appeared and developed. Previous studies (8–11) have found that the integrated operating room could reduce the interruption in the operation process, optimize the workflow. Thus, it was less time-consuming and potentially improved safety in the OR. Performing localization and operation in the same room is helpful to reduce the risks of adverse events during transferring. This study intends to evaluate the feasibility and efficiency of the integrated operating room and the non-intubated uniportal VATS in pulmonary nodules localization and resection.

## Materials and Methods

### Non-intubated Anesthesia

Localization and resection of pulmonary nodules were performed under non-intubated anesthesia. Propofol was administered intravenously via target-controlled infusion while using a mask to deliver oxygen and remove nitrogen (TCI). When the patient was successfully sedated, a laryngeal mask (WELL LEAD, Guangzhou, China) indo was inserted with a single dose of 0.05–0.1 mg/kg intravenous muscle relaxant (cisatracurium) during induction of anesthesia. The oxygenation was 40% at a rate of 3.5 mL/min. Following that, the continuous intravenous infusion of propofol through TCI, the remifentanil and dexmedetomidine hydrochloride were administered to maintain spontaneous breathing throughout the procedure. An intraoperative spectrum analyzer was utilized to monitor the depth of anesthesia during the procedure. The bispectral index (BIS) would be maintained between 40 and 60, while the respiratory rate would be between 12 and 18 beats per minute. To ensure the safety of the surgery, endotracheal intubation, thoracoscopic intubation, and mechanical breathing equipment were prepared to prevent airway blockage or severe hemorrhage.

### Navigation Procedure

**Figure 1:**
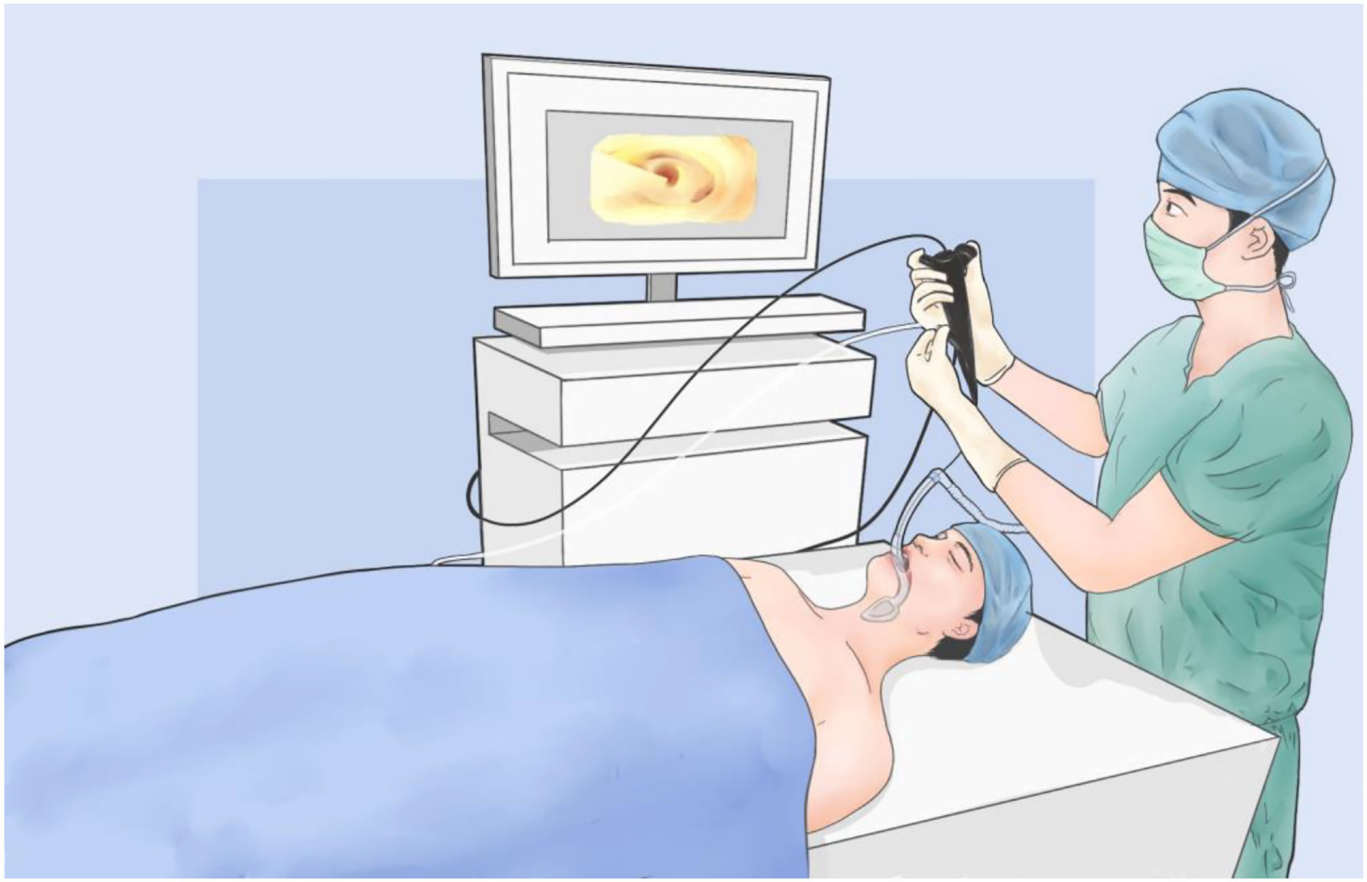
The diagram of electromagnetic navigation bronchoscopy **(**ENB) for pulmonary nodule localization.

The patient was given the entire intravenous anesthetic without endotracheal intubation in the integrated operating theater. The patient was in supine position on the electromagnetic bed during the navigation procedure. A bronchoscopy with the guidewire in the working channel was inserted through laryngeal mask. The ENB (LUNGCARE, Suzhou, China) has been placed along the navigation route into the target segmental bronchiole till the guidewire was calibrated the bilateral major bronchus and trachea (Figure 1). A dosage of indocyanine green (ICG, DANDONG YICHUANG, Liaoning, China) injections mixed with air was injected into the working channel for mapping. Once localization was completed, the patient would receive surgery directly in the same room.

### Uniportal VATS Procedure

**Figure 2:**
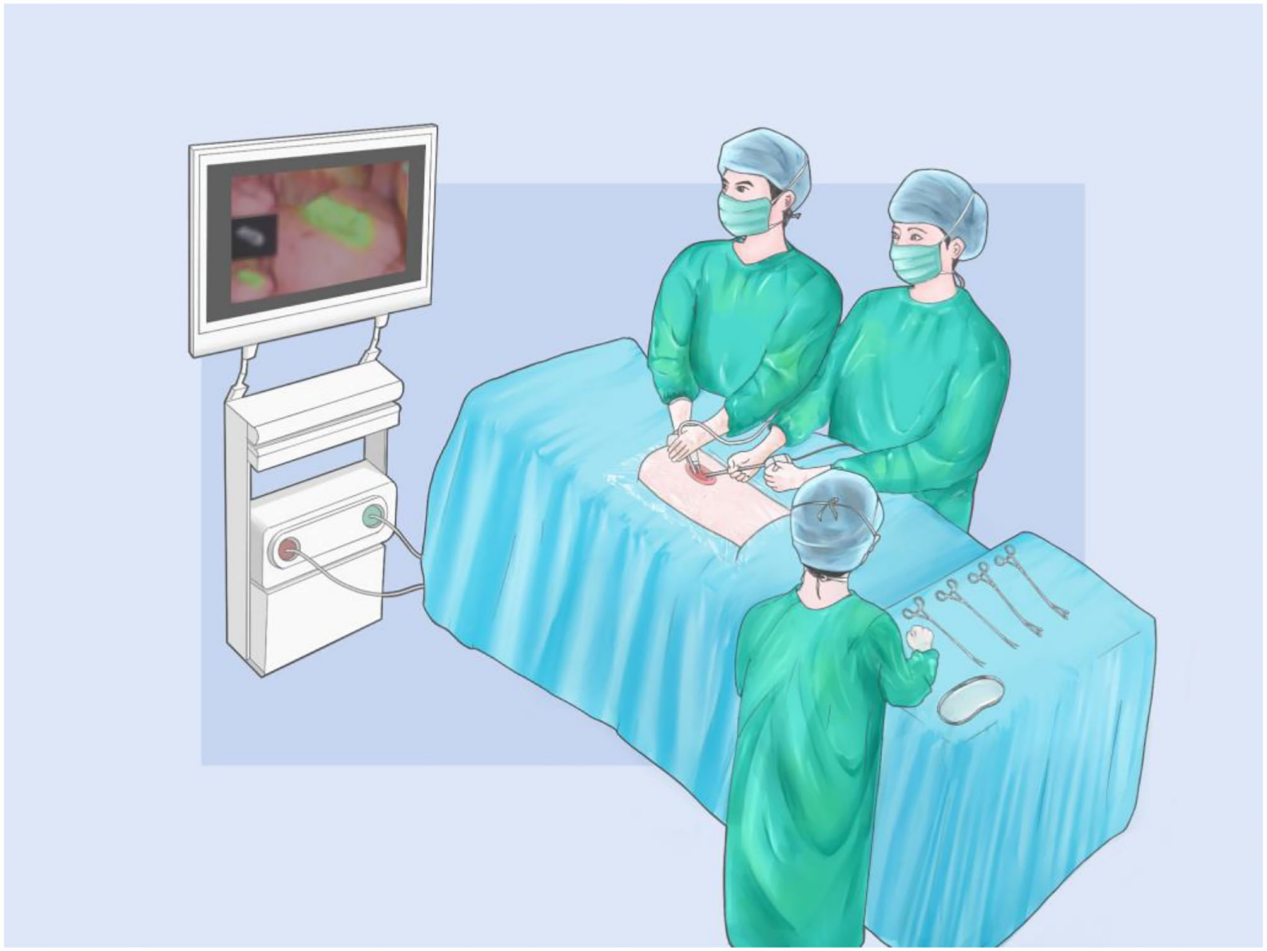
The diagram of uniportal NIVATS in subxiphoid approach.

Uniportal VATS was performed through a single incision at the fifth intercostal space or the subxiphoid (Figure 2). Supine position will be chosen when using a subxiphoid approach (nodules occurred in both left and right lobe), while a lateral position will be selected when nodules were ipsilateral. After introducing thoracoscopy, 2% lidocaine was sprayed on the lung surface and injected to block the sympathetic nerve to eliminate cough reflex and reduce the mediastinal movement. ICG fluorescence was detected by near-infrared thoracoscopy, and sublobectomy or lobectomy was performed according to frozen section results. After hemostasis and aerostasis were secured, the chest tube would be inserted through the incision and removed after the lungs were fully inflated. The incision would be sutured by layers.

### Patients

The inclusion criteria for this study were as follows: (a) preoperative thin-layer high-resolution CT scan confirmed the presence of pulmonary nodules; (b) ICG staining and localization of pulmonary nodules by ENB; (c) surgical history, including wedge resection, segmentectomy, and lobectomy; (d) the resection was under uniportal non-intubated VATS (NIVATS) in the integrated operating room; (e) the procedure performed by the same group of anesthesiologists and surgeons; (f) active follow-up after surgery.

### Data Analysis

Demographic data of the patients, including gender, age, height, weight, body mass index (BMI), and perioperative data, including calibration time, navigation time, resection range, postoperative complications, were collected and analyzed. Statistical analyses were performed using SPSS Statistic (Version 25, IBM Corp, Armonk, NY.).

## Results

### Patients

A total of 243 patients (Table 1) were enrolled from September 2018 to December 2021, including 88 males and 155 females. The average age was (52.32 ± 11.47) years old. The average body mass index (BMI) was (22.89 ± 2.94) kg/m^2^. Among them, 13 cases had a family history, 26 had a smoking history, 11 were positive for tumor markers, 212 had only one pulmonary nodule in CT examination, and 31 had multiple lesions (≥2 nodules). CT showed a total of 251 lesions, with an average diameter of nodules was (8.52 ± 4.64) mm. Most nodules were observed in the upper lobe and the least in the middle lobe (Table 2).

**Table 1:** Baseline characteristic (*n* = 243).

**Variables**	**Data**
**Gender**	
Male	88 (36.21%)
Female	155 (63.78%)
BMI (Mean ± SD, kg/m^2^)	22.89 ± 2.94
Age (Mean ± SD, yr)	52.32 ± 11.47
**Family history**	
Yes	13 (5.35%)
No	230 (94.65%)
**Smoking history**	
Yes	26 (10.70%)
No	217 (89.30%)
**Tumor marker**	
Positive	11 (4.53%)
Negative	232 (95.47%)
**Nodule number**	
1	212 (87.24%)
2	20 (8.23%)
3	6 (2.47%)
4	5 (2.06%)

**Table 2:** Nodules characteristic (*n* = 251).

**Variables**	**Data**
Nodule size (Mean ± SD,mm)	8.52 ± 4.64
**Location**	
Upper	148 (58.96%)
Middle	14 (4.58%)
Lower	89 (35.46%)
**Laterality**	
Left	107 (42.63%)
Right	144 (57.37%)
**Segments**	
S1+S2	110 (43.82%)
S3	31 (12.35%)
S4	16 (6.37%)
S5	8 (3.19%)
S6	24 (9.56%)
S7+S8	30 (11.95%)
S9	16 (6.37%)
S10	16 (6.37%)

### Operation-Related Results

**Figure 3:**
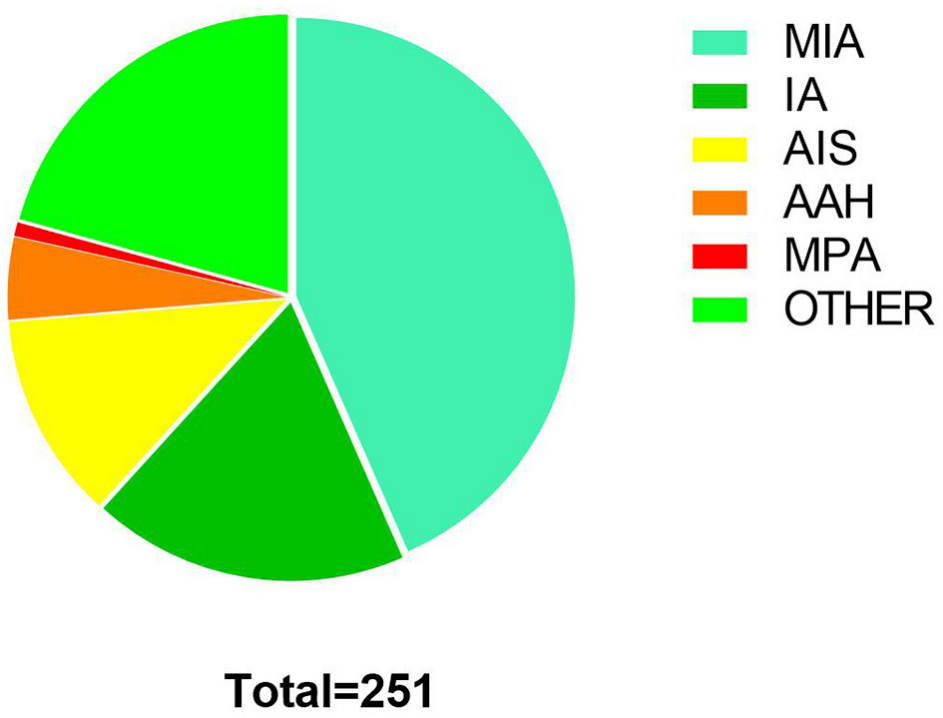
Pathological results of nodules: minimally invasive adenocarcinoma (MIA,109,43.43%), invasive adenocarcinoma (IA,46,18.32%), adenocarcinoma *in situ* (AIS,30,11.95%), atypical adenomatous hyperplasia (AAH,12,4.78%), mucin-producing adenocarcinoma (MPA, 2, 0.80%), other including metastatic tumor, pulmonary hamartoma, aspergillus nodules, tuberculoma, fibrous nodule, granuloma, et al. (52,20.72%).

Two hundred fifty-one pulmonary nodules (Table 2) underwent ICG staining localization by ENB between September 2018 to December 2021. The mean calibration time and navigation time, respectively, were 0.91 ± 0.43 min and 10.56 ± 7.24 min (Table 3). Overall, successful navigation occurred in 248 (98.80%) nodules, and the other three nodules were not localized successfully because of anatomical variation. Under the fluorescence control microscope, the fluorescence was located on the surface of the visceral pleura closest to the tumor, and no dye dissemination was found during the operation. There were no complications such as pneumothorax or pulmonary hemorrhage after localization. All patients received uniportal VATS after localization, including wedge resection (23,192.03%), segmentectomy (135.18%), and lobectomy (72.79%). The frozen sections were applied to all cases to identify the malignancies and to determine the resection margins. In this research, most of the nodules were diagnosed as minimally invasive adenocarcinoma (MIA, 109, 43.43%), and two nodules were mucin-producing adenocarcinoma (MPA, 2, 0.80%) (Figure 3). All nodules were completely resected without severe complications. The mean postoperative hospital was 1.80 ± 0.83 days, in which the majority was 2 days (122, 50.21%).

**Table 3:** Perioperative data.

**Variables**	**Data**
Calibration time (Mean ± SD, min)	0.91 ± 0.43
Navigation time (Mean ± SD, min)	10.56 ± 7.25
Successful navigation	248 (98.80%)
**Surgical mode**	
Wedge resection	231 (92.03%)
Segmentectomy	13 (5.18%)
Lobectomy	7 (2.79%)
**Postoperative hospital stays (d)**	
0	2 (0.82%)
1	89 (36.63%)
2	122 (50.21%)
3	14 (7.00%)
4	11 (4.53%)
5	2 (0.82%)

## Discussion

Lung cancer is the leading cause of cancer-related morbidity and fatality rate globally (12), and it also ranks top in China (13). The NCCN suggests that if there is a high suspicion of lung cancer, biopsy or surgical excision, with sufficient and adequate tissue sample to enable histology and molecular testing, are recommended (14). Moreover, trial No.JCOG0802 reported that segmentectomy would be a standard treatment for an NSCLC tumor diameter ≤ 2 cm if the superior pulmonary function and non-inferiority in overall survival are confirmed (15). Due to the advantages of less pain, shorter hospital stay, faster recovery, fewer complications, uniportal video-assisted thoracoscopic surgery has been recommended as the primary surgical therapy for pulmonary nodules (16, 17). However, accurate localization of pulmonary nodules is essential before surgery. Preoperative localization of pulmonary nodules now includes CT-guided hook-wire placement, nuclide or dye injection, intraoperative ultrasound localization, ENB dye injection, et.al. However, procedure-related complications, including pneumothorax, hemothorax, hematoma, and pleural reaction have been reported, which might jeopardize the postoperative quality of life and recovery to some extent. The rationale of this strategy is to enter the lung through the natural cavity and trachea to achieve nodules directly with the virtual scene. Localization of indocyanine green (ICG) injections under ENB can assist surgeons in identifying lung lesions more rapidly and precisely (18, 19). The ENB-guided ICG injection was utilized to localize pulmonary nodules in this report. Neither radiation nor displacement of the hook would appear in this method. Compared to the CT-guided localization, the dye would not spread in the thoracic cavity even if the patient's position was changed when dealing with the nodules near the visceral pleura. In addition, ICG staining has no impact on the pathological observation of the specimens, which would not jeopardize the clinical diagnosis. Moreover, the patients with multiple nodules would have to receive multiple radiations and punctures, suffering severe psychological pressure. For the three cases that were unsuccessfully localized by ENB because of anatomical variation, CT scan was performed and matched with individual 3D reconstruction before operation as the alternatives.

For the sake of minimal invasiveness, NIVATS was employed in this study. Double-lumen endotracheal intubation is required in the traditional VATS, which might induce airway trauma, ventilation lung damage, impair heart function (20), and even postoperative acute lung injury (ALI) (21). The emerging spontaneous breathing anesthetic can avoid direct harm from tracheal intubation. Lan L et al. reported that NIVATS showed a faster and more stable recovery in the post-anesthesia care unit (PACU), postoperative awaking, and PACU stay times were shorter. The use of sedatives and analgesics was less (22). Our previous preliminary feasibility study (16) also demonstrated that compared with the conventional VATS, patients who received spontaneous ventilation via uniportal VATS had a shorter postoperative recovery time, a shorter duration of postoperative hospitalization, and were able to eat and mobilize earlier. Also, patients receiving NIVATS had a less severe stress response, such as lower stress hormones including adrenaline, cortisol, and procalcitonin, and lower levels of inflammatory factors such as white blood cells, IL-6, IL-8, and CRP (5). In these cases, the patients whose perioperative hospital stay was from 0 to 2 days were 213 (87.65%) totally, which mirrored the result of studies mentioned above, showing that uniportal NIVATS could accelerate patient's recovery and improve patient's hospital experience.

None of the patients had a significant problem during or after the procedure. Compared to the traditional operating room, the integrated one is well-equipped that can fit most surgical operators' demands at any time. Unlike standard operating rooms, the integrated operating room allows for real-time focus imaging, which aids in proper diagnosis and treatment. The present study found that NIVATS for pulmonary nodules in an integrated operating room could enhance the efficiency and eliminate adverse events and inconveniences associated with the transferring. Jin et al. (23) reported similar findings that hybrid operating rooms successfully minimized thoracic surgical complications and mortality. This study suggested that such approach in the integrated room had a short mapping time, convenient operation, and high ENB localization technic success rate. Concerning the learning curve, it is easy for surgeons to get accustomed to the surgical vision and enhance hand-eye coordination in a shorter time (24). The concept of an integrated operating room combines the benefits of ENB and uniportal NIVATS, tailoring the diagnostic and treatment plan to the individual needs, providing a new option for personalized and precise diagnosis, treatment of pulmonary nodules, and improving postoperative rehabilitation.

However, limitations should be admitted in the present study. First, it is a retrospective single-arm study. Although the safety and feasibility were described in the study, the data was insufficient to demonstrate the superiority of the integrated operation room. Furthermore, integrated operating room has more stringent standards for operating equipment and medical personnel, making them unavailable for general practice. Therefore, it should be thoroughly considered before construction and application. Finally, the included patients are still in the follow-up process that the long-term outcomes are immature for analysis. Hence, implementing studies with a more rigorous design is warranted, including large-scale multicenter RCTs with more objective and clinically relevant outcomes.

## Conclusion

ENB localization and nodules resection under non-intubated uniportal VATS in the integrated operating room provide a feasible and efficient approach for pulmonary nodules, enhancing the treatment precision and postoperative recovery.

## Data Availability Statement

The original contributions presented in the study are included in the article/supplementary material, further inquiries can be directed to the corresponding authors.

## Ethics Statement

Ethical review and approval was not required for the study on human participants in accordance with the local legislation and institutional requirements. Written informed consent for participation was not required for this study in accordance with the national legislation and the institutional requirements.

## Author Contributions

This study was designed by SL and ZW and drafted by RW, YJ, and JH. YL contributed to collecting data. RW and ZW conducted the statistics analysis. All authors contributed to the article and approved the submitted version.

## Funding

This study was supported by National Key R&D Program of China (2017YFC0112704).

## Conflict of Interest

The authors declare that the research was conducted in the absence of any commercial or financial relationships that could be construed as a potential conflict of interest.

## Publisher's Note

All claims expressed in this article are solely those of the authors and do not necessarily represent those of their affiliated organizations, or those of the publisher, the editors and the reviewers. Any product that may be evaluated in this article, or claim that may be made by its manufacturer, is not guaranteed or endorsed by the publisher.

## References

[1] Bai C Choi C-M Chu CM Anantham D Ho JC Khan AZ. Evaluation of pulmonary nodules: clinical practice consensus guidelines for Asia. Chest. (2016) 150:877–93. 10.1016/j.chest.2016.02.650 26923625

[2] Leshnower BG Miller DL Fernandez FG Pickens A Force SD. Video-assisted thoracoscopic surgery segmentectomy: a safe and effective procedure. Ann Thorac Surg. (2010) 89:1571–6. 10.1016/j.athoracsur.2010.01.061 20417779

[3] Abbas AE. Surgical management of lung cancer: history, evolution, and modern advances. Curr Oncol Rep. (2018) 20:98. 10.1007/s11912-018-0741-7 30421260

[4] Wen Y Liang H Qiu G Liu Z Liu J Ying W. Non-intubated spontaneous ventilation in video-assisted thoracoscopic surgery: a meta-analysis. Eur J Cardiothorac Surg. (2020) 57:428–37. 10.1093/ejcts/ezz279 31725158

[5] Yu M Jing R Mo Y Lin F Du X Ge W. Non-intubated anesthesia in patients undergoing video-assisted thoracoscopic surgery: a systematic review and meta-analysis. PLoS ONE. (2019) 14:e0224737. 10.1371/journal.pone.0224737 31714904 PMC6850529

[6] Rodrigues JCL Pierre AF Hanneman K Cabanero M Kavanagh J Waddell TK. CT-guided microcoil pulmonary nodule localization prior to video-assisted thoracoscopic surgery: diagnostic utility and recurrence-free survival. Radiology. (2019) 291:214–22. 10.1148/radiol.2019181674 30720402

[7] Refai M Andolfi M Barbisan F Roncon A Guiducci GM Xiumè F. Computed tomography-guided microcoil placement for localizing small pulmonary nodules before uniportal video-assisted thoracoscopic resection. Radiol Med. (2020) 125:24–30. 10.1007/s11547-019-01077-x 31531810

[8] Buzink SN van Lier L de Hingh IHJT Jakimowicz JJ. Risk-sensitive events during laparoscopic cholecystectomy: the influence of the integrated operating room and a preoperative checklist tool. Surg Endosc. (2010) 24:1990–5. 10.1007/s00464-010-0892-6 20135171 PMC2895869

[9] Perrakis A Hohenberger W Horbach T. Integrated operation systems and voice recognition in minimally invasive surgery: comparison of two systems. Surg Endosc. (2013) 27:575–9. 10.1007/s00464-012-2488-9 22926891

[10] Pirlich M Stöhr M Neumuth T Dietz A. The intelligent ENT operating room of the future. Laryngorhinootologie. (2019) 98:S5–31. 10.1055/a-0751-3537 31096294

[11] Carstensen K Jensen EK Madsen ML Thomsen AML Løvschall C Tayyari Dehbarez N. Implementation of integrated operating rooms: how much time is saved and how do medical staff experience the upgrading? A mixed methods study in Denmark. BMJ Open. (2020) 10:e034459. 10.1136/bmjopen-2019-034459 32727737 PMC7394299

[12] Sung H Ferlay J Siegel RL Laversanne M Soerjomataram I Jemal A. Global cancer statistics 2020: GLOBOCAN estimates of incidence and mortality worldwide for 36 cancers in 185 countries. CA Cancer J Clin. (2021) 71:209–49. 10.3322/caac.21660 33538338

[13] Feng R-M Zong Y-N Cao S-M Xu R-H. Current cancer situation in China: good or bad news from the 2018 Global Cancer Statistics? Cancer Commun Lond Engl. (2019) 39:22. 10.1186/s40880-019-0368-6 31030667 PMC6487510

[14] Ettinger DS Wood DE Aisner DL Akerley W Bauman JR Bharat A. NCCN guidelines insights: non-small cell lung cancer, version 2.2021. J Natl Compr Cancer Netw JNCCN. (2021) 19:254–66. 10.6004/jnccn.2021.0013 33668021

[15] Suzuki K Saji H Aokage K Watanabe S-I Okada M Mizusawa J. Comparison of pulmonary segmentectomy and lobectomy: Safety results of a randomized trial. J Thorac Cardiovasc Surg. (2019) 158:895–907. 10.1016/j.jtcvs.2019.03.090 31078312

[16] Li S Jiang L Ang K-L Chen H Dong Q Yang H. New tubeless video-assisted thoracoscopic surgery for small pulmonary nodules. Eur J Cardio-Thorac Surg Off J Eur Assoc Cardio-Thorac Surg. (2017) 51:689–93. 10.1093/ejcts/ezw364 28007874

[17] Han D Cao Y Wu H Wang H Jiang L Zhao D. Uniportal video-assisted thoracic surgery for the treatment of lung cancer: a consensus report from Chinese Society for Thoracic and Cardiovascular Surgery (CSTCVS) and Chinese Association of Thoracic Surgeons (CATS). Transl Lung Cancer Res. (2020) 9:971–87. 10.21037/tlcr-20-576 32953478 PMC7481589

[18] Wang G. Lin Y Luo K Lin X Zhang L. Feasibility of injecting fluorescent agent under the guidance of electromagnetic navigation bronchoscopy in pulmonary nodule resection. Zhongguo Fei Ai Za Zhi Chin J Lung Cancer. (2020) 23:503–8. 10.3779/j.issn.1009-3419.2020.103.01 32517456 PMC7309554

[19] Kemp SV. Navigation bronchoscopy. Respir Int Rev Thorac Dis. (2020) 99:277–86. 10.1159/000503329 31600761

[20] Prisciandaro E Bertolaccini L Sedda G Spaggiari L. Non-intubated thoracoscopic lobectomies for lung cancer: an exploratory systematic review and meta-analysis. Interact Cardiovasc Thorac Surg. (2020) 31:499–506. 10.1093/icvts/ivaa141 32918464

[21] Umari M Falini S Segat M Zuliani M Crisman M Comuzzi L. Anesthesia and fast-track in video-assisted thoracic surgery (VATS): from evidence to practice. J Thorac Dis. (2018) 10:S542–54. 10.21037/jtd.2017.12.83 29629201 PMC5880994

[22] Lan L Cen Y Zhang C Qiu Y Ouyang B. A propensity score-matched analysis for non-intubated thoracic surgery.*Med Sci Monit Int Med J Exp Clin Res*. (2018) 24:8081–7. 10.12659/MSM.910605 30415268 PMC6410560

[23] Jin H Lu L Liu J Cui M. A systematic review on the application of the hybrid operating room in surgery: experiences and challenges. Updat Surg. (2021). 10.1007/s13304-021-00989-6. [Epub ahead of print]. 33709242

[24] Shi J He J He J Li S. Electromagnetic navigation-guided preoperative localization: the learning curve analysis. J Thorac Dis. (2021) 13:4339–48. 10.21037/jtd-21-490 34422360 PMC8339733

